# Comfort Analysis of Hafnium (Hf) Doped ZnO Coated Self-Cleaning Glazing for Energy-Efficient Fenestration Application

**DOI:** 10.3390/ma15144934

**Published:** 2022-07-15

**Authors:** Srijita Nundy, Aritra Ghosh, Abdelhakim Mesloub, Emad Noaime, Mabrouk Touahmia

**Affiliations:** 1 College of Engineering, Mathematics and Physical Sciences, Renewable Energy, University of Exeter, Penryn TR10 9FE, UK; s.nundy@exeter.ac.uk; 2 Department of Architectural Engineering, Ha’il University, Ha’il 2440, Saudi Arabia; a.maslub@uoh.edu.sa (A.M.); e.noaime@uoh.edu.sa (E.N.); 3 Department of Civil Engineering, Ha’il University, Ha’il 2440, Saudi Arabia; m.touahmia@uoh.edu.sa

**Keywords:** glazing, Hf-ZnO, building, g-value, *U*-value, glare, thermal comfort, visual comfort, CCT, CRI

## Abstract

To attain a comfortable building interior, building windows play a crucial role. Because of the transparent nature of the window, it allows heat loss and gain and daylight. Thus, they are one of the most crucial parts of the building envelope that have a significant contribution to the overall building energy consumption. The presence of dust particles on a window can change the entering light spectrum and creates viewing issues. Thus, self-cleaning glazing is now one of the most interesting research topics. However, aside from the self-cleaning properties, there are other properties that are nominated as glazing factors and are imperative for considering self-cleaning glazing materials. In this work, for the first time, Hf-doped ZnO was investigated as self-cleaning glazing and its glazing factors were evaluated. These outcomes show that the various percentages of ZnO doping with Hf improved the glazing factors, making it a suitable glazing candidate for the cold-dominated climate.

## 1. Introduction

Currently, buildings consume 40% of energy globally, which is due to the heating, ventilation and air conditioning load. This consumption has an adverse impact on the environment [1]. According to United Nations, migration from rural to urban areas is alarming and increasing every day. This urban influx also increases modern buildings’ energy consumption to maintain indoor comfort facilities [2,3,4]. Buildings generally consume high levels of energy due to their poorly thermally insulated envelopes [5]. Compared to other portions of envelopes, windows are critical, as they are the only parts of the building envelope that maintain the connection between the building’s interior and the exterior and allow daylight to penetrate [6].

The glazing sector is predominantly controlled by antireflection, self-cleaning and energy-saving, which are the key three principal functions [7]. For a hot climate, reflecting the solar heat or more precisely reflecting the NIR and IR part of the solar spectrum is the most strategic decision, which in turn reduces the air conditioning load [8,9]. However, antireflection is not suitable for cold climates as it is essential for the reflecting solar spectrum to be transmitted through the window to enhance the room temperature [10,11]. Hence, there is a trend now of replacing the traditional single- and double-glazed windows with advanced technology such as smart switchable EC [12], SPD [13,14,15,16], PDLC [17], thermally activated PCM [18], hydrogel [19,20], aerogel [21] or vacuum [22,23] filled windows.

The self-cleaning type of window is another class or area that can be applied to any type of building window (e.g., traditional and smart). Atmospheric pollutants possess significant viewing challenges for window glazing. Dust includes emissions from agriculture and industry, bird droppings, pollen, mineral dust in a dry area, fibers, sand and clay [24]. Daylight transmission into buildings is affected by the deposition of atmospheric pollutants on glazing [25]. Even in a clean UK climate, building windows suffer from dust [26]. Thus, a cleaned window is indispensable for a sustainable building. Depending on the particle diameter, they either fall from the glass surface or stick on the surface. Even though the glass surface looks smooth, it has microscopically small pocks which enhance the attraction of dirt [27,28,29]. A self-cleaning glazing or window is a thin self-cleaning coating of film on the external surface of the glass, which protects it from dirt [30]. Generally, two types of self-cleaning technologies are available: hydrophobic and hydrophilic. Self-cleaning glazing is capable of cleaning its own surface. For self-cleaning coating, transparency is essential, as it should not create any obstacles to indoor viewing. In addition, long-term durability is crucial for cost-effectiveness.

In the past, several self-cleaning materials have been investigated particularly for photovoltaic applications [31,32] and window [33,34] applications. Zinc oxide (ZnO) is one of the most bio-friendly important semiconductors that have been investigated for self-cleaning applications. A superhydrophobic ZnO nanorods@cellulose membrane for efficient building radiative cooling was investigated [35]. ZnO-coated transparent wood was employed for building applications previously and showed 17% energy saving compared to a traditional window [36]. In another work, a ZnO nanoparticle enhanced paraffin-filled window was investigated for double glazing which showed improved efficiency [37]. To further enhance the ZnO properties, Dy_2_WO_6_-doped ZnO [38], Sm_3+_-doped ZnO [39] and Hf-doped ZnO [40] have been investigated for self-cleaning. ZnO for self-cleaning is one of the most popular approaches [41].

Because of the similar ionic radii, Hf-doped ZnO has potential. Transition metal ion doping enhances the surface oxygen vacancies, which improves the self-cleaning behavior. The inclusion of lower-concentration hafnium increases oxygen vacancy defects and produces hydrophilic surfaces. Previously, hafnium oxide (HfO_2_) was prepared by electron beam evaporation, and three layers of HfO_2_/Ag/HfO_2_ showed heat mirror properties for energy-efficient window application [42]. We previously developed morphologically varied ZnO for self-cleaning application [43] and synthesized high-quality Hf-ZnO thin films with various Hf contents [40]. However, the suitability of ZnO in terms of glazing for building window applications has not yet been investigated.

How a new material will behave as a building window can be understood by analyzing its thermal and visual comfort parameters [44,45]. The solar heat gain coefficient or solar factor is one of the major influential factors that determine the indoor room temperature and thus define the thermal comfort level [46]. Most often occupants prefer a 20 °C temperature in indoor conditions [47,48]. For a cold climate, a higher solar factor is essential as it increases the room temperature and maintains a comfortable level, whereas for a hot climate, the solar factor should be limited or rejected to limit the increase in room temperature [49,50]. Visual comfort includes both the illuminance level and the color properties. Bright ambient daylight is paramount for cognitive work [51]. However, this amount should not exceed a certain level, or else discomfort glare will dominate. External daylight transmitted through the window glazing attains wavelength changes, which can create discomfit for occupants [52,53,54]. Color property analysis tackles these challenges.

In this work, for the first time, Hf-doped ZnO was investigated for glazing application. Thus, to understand its suitability as a future self-cleaning fenestration, glazing factor and thermal and visual comfort analyses are essential. Employing the measured transmission spectrum of different Hf-doped ZnO, essential glazing factors such as solar and luminous transmission, solar material protection factor (SMPF) and solar skin protection factors (SSPFs) have been calculated. For thermal comfort analysis, the solar factor has been evaluated. Further, correlated color temperature (CCT), color rendering index (CRI) and glare have also been calculated to understand the visual comfort and suitability of this material for building fenestration application.

## 2. Experiments

### 2.1. Materials Fabrication for Glazing

**Figure 1:**
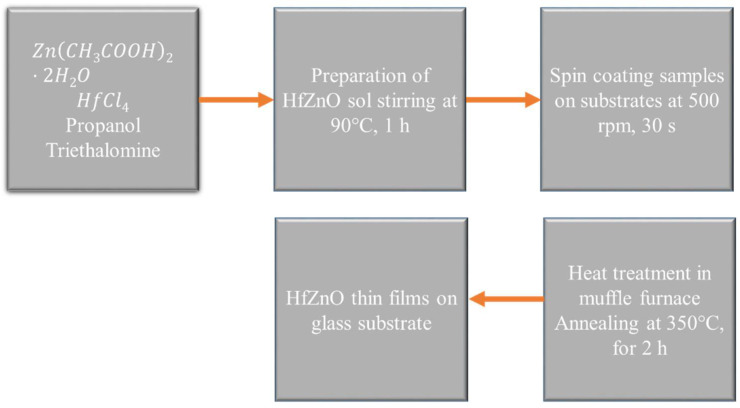
Schematic illustration of involved steps for synthesis of hafnium-doped ZnO.

The material for the self-cleaning glazing purpose was developed using hafnium IV chloride (HfCl_4_), propanol (C_2_H_5_OH), triethanolamine (C_6_H_15_NO_3_) and zinc acetate (Zn(CH_3_COOH)2·2H_2_O), which were purchased from Sigma Aldrich (St. Louis, MI, USA) and used without any further purification. Pure and Hf-doped ZnO were synthesized using the sol–gel synthesis method with Hf concentrations varying from 0 to 15%. Briefly, 2.2 g of Zn(CH_3_COOH)2·2H_2_O was made to dissolve completely in 10 mL of C_2_H_5_OH. Then, C_6_H_15_NO_3_ was carefully poured into the above-prepared solution, where the molar ratio of triethanolamine:zinc acetate was kept at 3:5. The resultant mixture was maintained at room temperature for 5 min. Part of this sol was directly taken for preparation of pure ZnO, and the rest was separated into batches, wherein a particular amount of HfCl_4_ was added, and stirred at 90 °C for 1 h, thereby forming the sol for Hf-doped ZnO. These as-prepared sols were taken for the thin-film coating on glass substrates, via spin coating (Ossila spin coater, Sheffield, UK), carried out at 500 rpm for 30 s. The as-deposited thin films were taken for annealing in a muffle furnace at 350 °C for 2 h. Finally, the pure and Hf-doped ZnO thin films on glass substrates were obtained after cooling down to room temperature and were further taken for characterization and application purposes. Figure 1 shows the schematic representation of different involved steps for the synthesis of the material.

### 2.2. Optical Characterization

For optical characterization of the developed glazing, a PerkinElmer Lambda 1050 spectrometer (Waltham, MA, USA) which could measure the visible and NIR transmission and reflection was employed. This system had a 150 nm diameter-based integrating sphere, and measurement was carried out at 10 nm intervals.

## 3. Methods

### 3.1. Glazing Factor Evaluation

Solar and luminous transmittance was evaluated by employing Equations (1) and (2), respectively. *T*(*λ*) is the spectral transmission of glazing. The relative spectral distribution of the illuminant is D_65_, *S*(*λ*) is the relative spectral distribution of solar radiation, *V*(*λ*) is the spectral luminous efficiency of a standard photopic observer, and wavelength interval is represented by Δ*λ*.

Protection factors are crucial building window parameters that show the ability of a window to protect the building material and human skin (located behind the window) when they are exposed to solar radiation [55]. The solar material protection factor (*SMPF*) is associated with the protection of building material, and the solar skin protection factor (*SSPF*) is associated with the human skin [56]. *SMPF* and *SSPF* both vary between 0 and 1 [57]. Values close to 0 indicate a low protection level, whereas close to 1 indicate a high protection level. *SMPF* and *SSPF* are represented by Equations (3) and (4).

Solar transmission

(1) $$\tau_{s}=\frac{\sum_{\lambda \;=\;300\;\mathrm{nm}}^{2500\;\mathrm{nm}}S\left(\lambda \right)T\left(\lambda ,\alpha \right)\Delta \lambda }{\sum_{\lambda \;=\;300\;\mathrm{nm}}^{2500\;\mathrm{nm}}S\left(\lambda \right)\Delta \lambda }$$

Luminous transmission

(2) $$\tau_{v}=\frac{\sum_{\lambda \;=\;380\;\mathrm{nm}}^{780\;\mathrm{nm}}D_{65}\left(\lambda \right)T\left(\lambda ,\alpha \right)V\left(\lambda \right)\Delta \lambda }{\sum_{\lambda \;=\;380\;\mathrm{nm}}^{780\;\mathrm{nm}}D_{65}\left(\lambda \right)V\left(\lambda \right)\Delta \lambda }$$

Solar material protection factor (*SMRF*)

(3) $$SMRF=1-\frac{\sum_{\lambda \;=\;300\;\mathrm{nm}}^{600\;\mathrm{nm}}T\left(\lambda \right)C_{\lambda }S_{\lambda }\Delta \lambda }{\sum_{\lambda \;=\;300\;\mathrm{nm}}^{600\;\mathrm{nm}}C_{\lambda }S_{\lambda }\Delta \lambda }$$

where $C_{\lambda }=e^{-0.012\lambda }$.

Solar skin protection factor (*SSPF*)

(4) $$SSPF=1-\frac{\sum_{\lambda \;=\;300\;\mathrm{nm}}^{400\;\mathrm{nm}}T\left(\lambda \right)E_{\lambda }S_{\lambda }\Delta \lambda }{\sum_{\lambda \;=\;300\;\mathrm{nm}}^{400\;\mathrm{nm}}E_{\lambda }S_{\lambda }\Delta \lambda }$$

*E_λ_* is the CIE erythemal effectiveness spectrum.

### 3.2. Thermal Comfort

The amount of solar energy transmitted through the transparent and semitransparent part of the window is represented by the solar heat gain coefficient or solar factor (*g*). This includes entering infrared radiation into a building’s interior and solar transmittance [55,57].

(5) $$\begin{array}{cc} g & =\tau_{s}+q_{i}=\tau_{s}+\alpha \frac{h_{i}}{h_{i}\;+\;h_{e}}\\ & =\tau_{s}+\left(1-\tau_{s}-\rho_{s}\right)\frac{h_{i}}{h_{i}\;+\;h_{e}} \end{array}$$

where *h_e_* and *h_i_* are the external and internal heat transfer coefficients.

### 3.3. Visual Comfort

Quality and quantity of light in indoor conditions are essential to understanding and analyzing visual comfort. Correlated color temperature (*CCT*) and color rendering index (*CRI*) both indicate the quality of indoor daylight [58]. Compared to external daylight, *CRI* shows the rendering ability of the incoming daylight. *CCT* is measured in kelvin (*K*) and signifies a light source’s “coolness” and “warmth”. *CRI* over 80 is accepted for building window application, and *CRI* over 90 is outstanding [59,60,61]. For *CCT*, the range between 3000 K and 7500 K is desired for transmitted daylight.

*CCT* was calculated from McKamy’s equation [62].

(6) $$CCT=449n^{3}+3525n^{2}+6823.3n+5520.33$$

where $n=\frac{\left(x\;-\;0.3320\right)}{\left(0.1858\;-\;y\right)}$ and *x* and *y* are chromaticity coordinates.

The color rendering index (*CRI*) is given by

(7) $$CRI=\frac{1}{8}\sum_{i=1}^{8}R_{i}$$

The total distortion Δ*E_i_* is determined from

(8) $$\Delta E_{i}=\sqrt{{\left(U_{t,i}^{*}-U_{r,i}^{*}\right)}^{2}+{\left(V_{t,i}^{*}-V_{r,i}^{*}\right)}^{2}+{\left(W_{t,i}^{*}-W_{r,i}^{*}\right)}^{2}}$$

The special color rendering index *R_i_* for each color sample is given by

(9) $$R_{i}=100-4.6\Delta E_{i}$$

**Figure 2:**
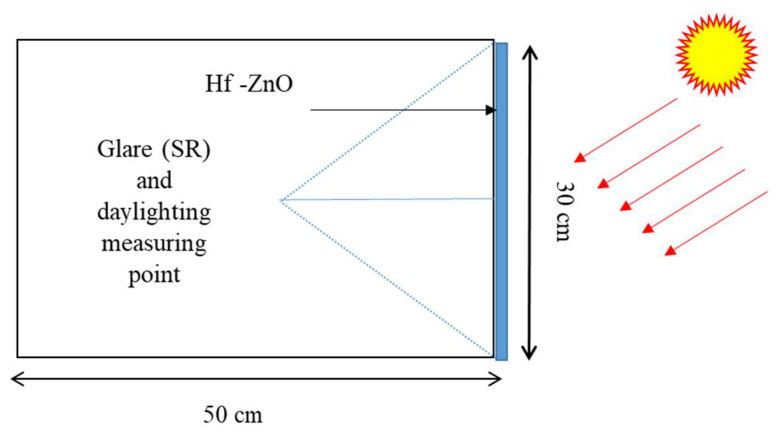
Schematic cross-section of a room with perovskite glazing mounted on vertical south facade.

To understand the quality of the indoor light, daylight glare evaluation is essential; daylight glare was evaluated in this work by employing glare subjective rating (*SR*) (as shown in Equation (10)) [63]. Minimum engagement of photosensors makes this method widely available and useful because it saves time and cost [64]. Theoretically, glare control potential using this glazing was identified from measured outdoor illuminance on a vertical plane as shown in Figure 2. This *SR* index allows the estimation of discomfort glare experienced by subjects when working at a visual daylight task (VDT) placed against a window of high or non-uniform luminance.

(10) $$SR=0.1909E_{v}^{0.31}$$

*SR* for a typical sunny day in the cold-dominated climate of Penryn, UK (50.16° N, 5.10° W), was examined. Vertically south-facing Hf-doped ZnO glazing having dimensions of 30 × 30 × 0.5 (l × *w* × *h*) cm in the scale model was considered, as shown in Figure 2. This large area resembles self-cleaning glazing as a large facade, while the internal surface was painted in white color with a reflectance of 0.8 [65]. Internal vertical illuminance (*E_V_*) facing the window (worst case) was measured at the center of the room. Table 1 displays the criterion scale of *SR*. This method also allows the non-intrusive measuring equipment necessary for scale model daylighting assessments [66,67].

## 4. Results

### 4.1. Optical Transmission

**Figure 3:**
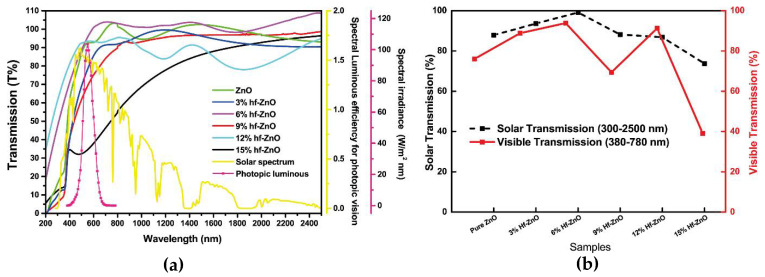
(**a**) Wavelength-dependent UV, visible and NIR transmission spectra of pure and Hf-doped ZnO thin films. (**b**) Relation between solar and visible transmission for ZnO with various levels of Hf doping.

Figure 3a shows the spectral transmission of various Hf-doped ZnO for the wavelength range between 250 and 2500 nm. Transmission dropped for 15% Hf doping, while the highest transmission was observed for 6% Hf doping. The product of the spectral luminous efficiency for photopic vision *V*(*λ*) and relative spectral distribution of illuminant D65(*λ*) has been included for comparison; it varied from 400 nm to 700 nm, having its peak at 555 nm. Figure 3b shows the comparison of single value solar and visible transmission for pure and different Hf-doped ZnO. Pure ZnO showed 87% solar transmission, while 3%, 6%, 9%, 12% and 15% showed 87%, 99%, 88%, 86% and 73%, respectively. Extraordinary changes occurred while the Hf doping percentage was 6%. Visible transmissions for pure and different Hf-doped ZnO are 75% (pure), 88% (3%), 93% (6%), 69% (9%), 91% (12%) and 39% (15%).

**Figure 4:**
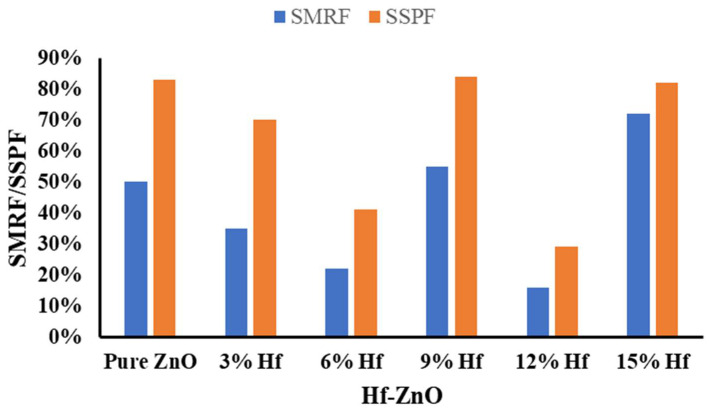
Solar material protection factor (*SMRF*) and solar skin protection factor (*SSPF*) for pure and different Hf-doped ZnO.

Figure 4 illustrates the solar material protection factor and skin protection factor for pure and different Hf-doped ZnO. The material protection factor was higher for 15% Hf-doped ZnO, which was the reason for its lower transmission. Less solar transmission indicates lower degradation. The skin protection factor was lowest for the 12% Hf-doped ZnO, which was due to its highest transmission.

### 4.2. Comfort Analysis

**Figure 5:**
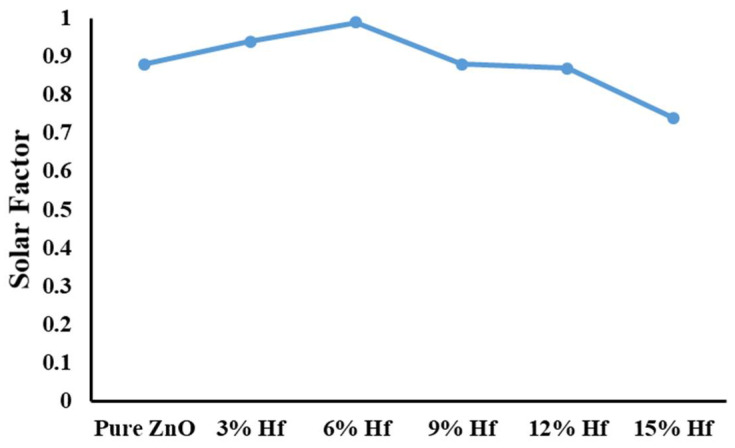
Solar factor for self-cleaning glazing based on different Hf-doped ZnO.

Figure 5 illustrates the solar factor for self-cleaning glazing based on different Hf-doped ZnO. The solar factor is a crucial element for building glazing as its presence is highly recommended for a cold climate, whereas its rejection is essential for a hot climate. In this work, 6% Hf-doped ZnO showed the best solar factor for the cold-dominated climate. However, if this glazing is adopted in a heat-dominated climate, 15% Hf-doped ZnO should be selected. High values of solar factors indicate that the reflection of solar radiation from these glazings is minimal. This is also aesthetic as high reflection can cause issues for the other building users.

**Figure 6:**
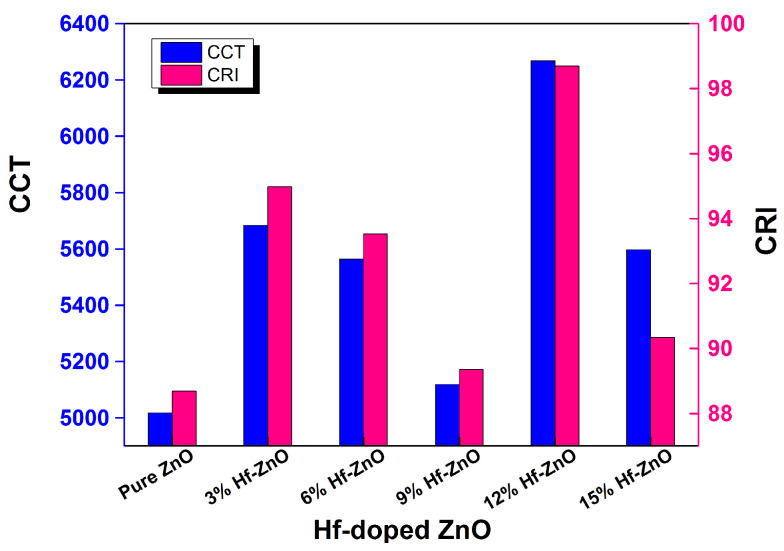
Color rendering index (*CRI*) and correlated color temperature (*CCT*) of pure and Hf-doped ZnO thin films.

Color properties, including *CCT* and *CRI*, were calculated for Hf-ZnO glazing using Equations (6) (*CCT*) and (7) (*CRI*) and are shown in Figure 6. The 12% Hf-doped ZnO had the best CRI (>98) and CCT (>6200). Interestingly all the doped ZnO samples had higher *CRI* than the pure ZnO. These values satisfy the acceptance level for the comfort level criteria as prescribed in CIE *CIR* [68,69] and IES TM 30–15 [70]. In addition, it can be proposed that *CRI* and *CCTs* are not dependent on a single transmittance value, but their dependency relies on the overall spectrum range. A very similar outcome was previously demonstrated for other types of glazing [71,72].

**Figure 7:**
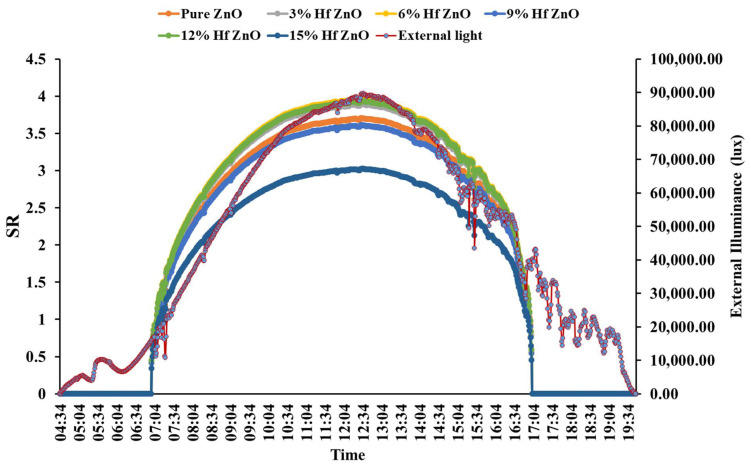
Glare (subjective rating) for pure and different Hf-doped ZnO.

Figure 7 shows the *SR* for different Hf-doped ZnO and pure ZnO-based glazing for a vertical south-facing large glazed facade located in cold-dominated climate of Penryn, in the southwest of the UK. A typical clear sunny day was considered for this analysis. The location of the subject is shown in Figure 2. It is clear from the figure that except for the 15% Hf-doped ZnO, others were not able to maintain the glare. This is definitely due to the high transmission rate for all the different Hf-doped and pure ZnO-based glazings. For a cold climatic country where the heating load is high, this penetration of higher solar light could be beneficial from a thermal comfort point of view, although visual comfort may be compromised. However, this argument is true for any type of window for which it is not possible to attain visual and thermal comfort concomitantly. The promising factor for this type of coating is a high transmission, which is key for any self-cleaning material. Transmission reduction on a double glass due to self-cleaning coating is not at all acceptable. Except for building windows, this analysis also strongly recommended the use of this material for self-cleaning coating for the PV system as no transmission reduction is attained and mostly very high transmission was achieved, particularly for the 3%, 6% and 12% Hf-doped ZnO.

## 5. Conclusions

In this work, glazing factors and thermal and visual comfort analyses of a self-cleaning coated glazing were examined. This particular self-cleaning coating was developed by the sol–gel method with the introduction of 3%, 6%, 9%, 12% and 15% Hf doping of ZnO. Results of these doped ZnO samples were also compared with pure ZnO. The visible transmission was always higher for the 6% doped ZnO. The protection factor had no trend with an increase in Hf doping. The lowest protection factor was observed at 12% Hf doping. CRI’s threshold value of 80 was achieved for all the Hf-doped ZnO type glazings. A higher amount of solar factor also makes this glazing suitable for cold-dominated climates. This high solar factor also indicates that the glazing possesses lower reflection. The 15% doped ZnO showed an allowable SR limit compared to other doped ZnO samples. This was due to the lowest transmission level at the visible wavelength for 15% doped ZnO. This self-cleaning glazing can be a solution for future energy-efficient window applications. Particularly for cold climate conditions, this self-cleaning can be a good candidate for building window application because of its high solar and visible transmission and high solar factor. In addition, because of lower reflection, it can also be applied on top of photovoltaic systems to diminish the soiling issues. In the future, further investigation is required to understand the reliability of this coating under real weather conditions after long-term outdoor exposure (following different Köppen climatic conditions).

## Figures and Tables

**Table 1:** Criterion scale of discomfort glare subjective rating (SR) [63].

Comfort Level Indicator	Glare Subjective Rating (*SR*)
Just intolerable	2.5
Just disturbing	1.5
Just noticeable/acceptable	0.5

## Data Availability

Not applicable.
